# Land use mix and leukocyte telomere length in Mexican Americans

**DOI:** 10.1038/s41598-021-99171-6

**Published:** 2021-10-05

**Authors:** Hua Zhao, Jie Shen, David Chang, Yuanqing Ye, Xifeng Wu, Wong-Ho Chow, Kai Zhang

**Affiliations:** 1 Department of Family Medicine and Population Health, School of Medicine, Virginia Commonwealth University, 830 East Main Street, Richmond, VA 23298 USA; 2 Empire Innovation Associate Professor, Department of Environmental Health Sciences, School of Public Health, University at Albany, State University of New York, Rensselaer, NY USA; 3 Department of Epidemiology, The University of Texas MD Anderson Cancer Center, Houston, TX USA

**Keywords:** Environmental sciences, Environmental social sciences, Risk factors

## Abstract

It has been well-known that built environment features influence the risk of chronic diseases. However, the existing data of its relationship with telomere length, a biomarker of biological aging, is still limited, with no study available for Mexican Americans. This study investigates the relationship between several factors of the built environment with leukocyte telomere length among 5508 Mexican American adults enrolled in Mano-A-Mano, the Mexican American Cohort Study (MACS). Based on the quartile levels of telomere length, the study population was categorized into four groups, from the lowest (1st quartile) to the highest telomere length group (4th quartile). For individual built environment factors, their levels did not differ significantly across four groups. However, in the multinominal logistic regression analysis, increased Rundle’s land use mixture (LUM) and Frank’s LUM were found statistically significantly associated with increased odds of having high levels of telomere length (Rundle’s LUM: 2nd quartile: Odds ratio (OR) 1.26, 95% Confidence interval (CI) 1.07, 1.48; 3rd quartile: OR 1.25, 95% CI 1.06, 1.46; 4th quartile: OR 1.19, 95% CI 1.01, 1.41; Frank’s LUM: 2nd quartile: OR 1.34, 95% CI 1.02, 2.63; 3rd quartile: OR 1.55, 95% CI 1.04, 2.91; 4th quartile: OR 1.36, 95% CI 1.05, 2.72, respectively). The associations for Rundle’s LUM remained significant after further adjusting other non-redundant built environment factors. Finally, in stratified analysis, we found the association between Rundle’s LUM and telomere length was more evident among younger individuals (< 38 years old), women, and those with obesity, born in Mexico, having low levels of physical activity, and having low levels of acculturation than their relative counterparts. In summary, our results indicate that land use mixture may impact telomere length in leukocytes in Mexican Americans.

## Introduction

It is estimated that by 2050, 70% of the projected world population will live in urban areas^1^. Urban living comes with multiple health challenges, including decreasing physical activity levels and increased levels of obesity and obesity-related disease^2–6^. Thus, finding the urban built environment features that promote people living an active lifestyle and consequently improve overall health is critically needed. Studying the built environment is particularly relevant to Mexican Americans^7,8^. Because of their lower socioeconomic status, they are more likely to have health outcomes that are more susceptible to variations in the built environment than Whites^9^. In our previous study in Mano-A-mano, the Mexican American Cohort study (MACs), we found that less than 50% and 25% of all men and women met US physical activity recommendations^10^. Mexican Americans living in areas with a high density of roads had higher body mass index (BMI) than their counterparts^11^. We also observed that selected built environment factors (e.g., population density and road/intersection ratio) affect leukocyte mitochondrial DNA copy number levels, a biomarker linking environmental exposures and chronic diseases^12^.

In addition to mitochondrial DNA copy number, telomere length is another biomarker thought to connect environmental exposures and chronic diseases. Telomeres are the repeat elements at the ends of DNA that protect chromosomes and shorten with each replicative cycle. When they reach a critically short stage, the cell goes into a state of senescence or apoptosis. It has been suggested that telomere length in leukocytes provides insight into immune system health. Maintain an active lifestyle is known to strengthen the immune system and reduce systemic inflammation^13–15^. Our previous study in Mexican Americans reported a reverse association between leukocyte telomere length and sitting time^16^. Altered telomere length has also been linked to multiple health conditions that exhibit an association with short telomere lengths, such as hypertension^17^, cardiovascular disease^18^, diabetes^19^, obesity^16,20^, and cancers^21^. Though several existing studies have shown that social environment features and neighborhood socioeconomic status affected telomere length^22–31^, few studies have assessed the role of the built environment. In a cross-sectional study of 1488 individuals, inverse associations between telomere length with population density and urban crowding were reported^28^. However, none of those existing studies focus on Mexican Americans, who are susceptible to changes in the built environment.

In this study, we attempted to estimate the associations between several factors of the built environment (e.g., physical activity environment, land use, and food environment) and leukocyte telomere length among adult participants enrolled in the MAC study, a large Mexican American cohort study in the U.S.^32^. We hypothesized that exposure to certain built environment features might alter telomere length.

## Methods

### Study population

We identified the study participants from those registered in the MAC study, a large population-based cohort of Mexican-origin households recruited in the Houston, Texas area. A detailed report of the MAC study has been published before^32^. A total of 5508 participants from the MAC study were included in this study. The inclusion criteria included: age > 20 years old; no reported cancer, diabetes, or cardiovascular diseases at the time of cohort entry; and measured data on leukocyte telomere length. The Institutional Review Board of the MD Anderson Cancer Center approved the study. All methods were performed following the relevant guidelines and regulations. Written informed consent has been obtained from all participants.

### Measurement of leukocyte telomere length

The exact method and data are described in our previous publication^16^. We used a modified version of the real-time quantitative polymerase chain reaction (PCR) method initially described by Cawthon^33^ to measure leukocyte telomere length. Briefly, the ratio of the telomere repeat copy number (T) to the single gene (human globulin) copy number (S) was determined for each sample. The derived T/S ratio was proportional to the overall telomere length. The PCR reaction mixture for the telomere amplification consisted of 1 × SYBR Green Master Mix (Applied Biosystems, Foster City, CA, USA), 200 nmol/l Tel-1 primer (5′-CGGTT TGTTTGGGTTTGGGTTTGGGTTTGGGTTTGGGTT), 200 nmol/l Tel-2 primer (5′-GGCTT GCCTTACCCTTACCCTTACCCTTACCCTTACCCT), and 5 ng genomic DNA. Similarly, the PCR reaction mixture (14 µl) for *HGB* gene amplification consisted of 1 × SYBR Green Master Mix, 200 nmol/l Hgb-1 primer (5′-GCTTCTGACACAACTGTGTTCACTAGC), 200 nmol/l Hgb-2 primer (5′-CACCAA CTTCATCCACGTTCACC), and 5 ng genomic DNA. The thermal cycling conditions were 1 cycle at 95 °C for 10 min followed by 40 cycles at 95 °C for 15 s and at 56 °C (for telomere amplification) or 58 °C (for *HGB* amplification) for 1 min. Each sample was run in duplicate in a 384-well plate. The telomere and *HGB* PCRs were done on separate 384-well plates with the same samples in the same well positions. In each run, negative and positive controls, a calibrator DNA, and a standard curve were included. The positive controls contained a telomere of 1.2 kb and a telomere of 3.9 kb from a commercial telomere length assay kit (Roche Applied Science, Pleasanton, CA, USA). For each standard curve, 1 reference DNA sample (the same DNA sample for all runs) was diluted twofold serially to produce a 6-point standard curve between 20 ng and 0.625 ng of DNA in each reaction. The coefficient of determination (R^2^) for each standard curve was ≥ 0.99, with acceptable standard deviations set at 0.25 (for the Ct values). The intra-assay coefficient of variation was < 3%, and the inter assay coefficient of variation was < 5% for RTL assay in our laboratory. All 5508 study subjects passed the quality control so all of them were included in the further analysis.

### Built environment factors

The data on built environment factors are presented in our previous publication^11,12^. In brief, to represent essential areas of the built environment, we assessed physical activity environment, land use variety, and food environment., Five exposure variables [population density (people per km^2^), household density (households per km^2^), road density (number of road links per km^2^), and intersections density (number of road intersections per km^2^)] within a half-mile radius surrounding each participant's residence were included in the physical activity environment. We also included the nearest parks and the highways around each participant's residential address. We used two indexes of land use mix for land use based on two published methods by Rundle et al.^34^ and Frank et al.^35^. For both indexes, values closer to 1 indicate highly diverse land use within a half-mile buffer in our study, whereas values closer to 0 indicate that one land use type is dominant. Rundle's LUM considered two types of land use—residential and commercial uses. Frank's LUM considered multiple types of land use. Besides residential and commercial uses, it also included farm ranch, industrial, parks, underdeveloped, and vacant areas in our study based on available land use features. The food environment was assessed as the weighted Modified Retail Food Environment Index (mRFEI) published by the Centers for Diseases Control and Prevention (CDC).

### Statistical analysis

Log transformed telomere length was used in the analysis. Based on the quartile levels of telomere length, the study population was categorized into four groups, from the lowest (1st quartile) to the highest telomere length group (4th quartile). Descriptive statistics [e.g., mean and standard deviation (S.D.)] were applied to each demographic and built environmental variable in each category. ANOVA was applied to assess the difference across four categories. We used pair-wise correlation analysis to identify the relationships among built environment variables. For each built environment variable, we used the multinominal logistic regression analysis to evaluate its association with telomere length, while four quartile levels of telomere length were used as the outcome variable. The covariates included in the model were selected backward stepwise selected. They were age (continuous), sex (men vs. women), BMI (continuous), physical activity (high, median, vs. low), health insurance (yes vs. no), born place (Mexico vs. U.S.), acculturation (low vs. high), and census income (continuous) as covariates. For each built environmental variable, we assessed its association with the telomere length using odds ratios (ORs) and 95% confidence intervals (CIs). To further assess the possible joint effect, we included selected non-redundant built environment variables simultaneously in multinominal logistic regression. A similar multinominal logistic regression analysis was also applied in the stratified analysis to identify a difference by selected demographic variables. All statistical analyses were conducted in SAS software (version 9.3; SAS Institute Inc., Cary, NC, USA). A two-sided P-value < 0.05 was required for statistical significance.

### Ethics approval and consent to participate

The study was approved by the Institutional Review Board of MD Anderson Cancer Center. All methods were performed in accordance with the relevant guidelines and regulations. Written consent has been obtained from all participants.

## Results

We split the study population (n = 5508) into four groups using the quartile levels of telomere length. Table 1 showed the basic demographics of each group. With the telomere length increased from group 1 to group 4, significantly decreasing age and census income trends were observed (P < 0.001 and P = 0.014, respectively). Also, women and those with high levels of acculturation were more likely to be in the higher telomere length group (higher telomere length) compared to their counterparts (P < 0.001 and P = 0.024, respectively). BMI, place of birth, insurance status, physical activity did not differ by telomere length category. We included ten built environment variables, including population density, household density, intersection density, road density, distance to highway, walking time to the nearest park, networked distance to the nearest park, Rundle’s LUM, Frank’s LUM, and CDC mRFEI. The respective mean levels and SD in each telomere length group are listed in Table 1. No significant association was observed when they were compared across the telomere length groups.

**Table 1:** Description of built environment variables covariates of in quartile levels of telomere length.

Variables	1st quartile (N = 1354)	2nd quartile (N = 1393)	3rd quartile (N = 1377)	4th quartile (N = 1384)	P value
	Mean (SD)	Mean (SD)	Mean (SD)	Mean (SD)	
Population density	2.42 (0.90)	2.3 7 (0.94)	2.38 (0.93)	2.42 (0.91)	0.970
Household density	0.83 (0.35)	0.81 (0.35)	0.81 (0.35)	0.82 (0.34)	0.304
Intersection density	3.12 (0.97)	3.08 (0.96)	3.12 (1.00)	3.12 (0.94)	0.630
Road density	5.75 (1.87)	5.69 (1.84)	5.76 (1.92)	5.76 (1.80)	0.691
Distance to highway	1.14 (0.87)	1.15 (0.91)	1.12 (0.83)	1.14 (0.87)	0.719
Walking time to the nearest park	11.07 (9.62)	11.32 (9.71)	11.31 (0.51)	10.70 (8.83)	0.291
Networked distance to the nearest park	0.90 (0.79)	0.92 (0.83)	0.91 (0.78)	0.86 (0.72)	0.215
Rundle's LUM	0.44 (0.20)	0.49 (0.33)	0.49 (0.33)	0.48 (0.24)	0.088
Frank's LUM	0.57 (0.08)	0.58 (0.08)	0.58 (0.08)	0.59 (0.08)	0.183
CDC mREFI	8.61 (4.93)	8.55 (4.72)	8.34 (4.59)	8.80 (4.64)	0.495
Age	41.66 (13.18)	41.05 (12.41)	40.34 (12.65)	36.29 (11.29)	< 0.001
BMI	31.37 (6.34)	31.52 (6.66)	31.71 (6.77)	31.32 (6.72)	0.350
Census income	$37,995 ($13,359)	$37,970 ($14,000)	$37,412 ($12,959)	$36,491 ($12,651)	0.014

	Number (%)	Number (%)	Number (%)	Number (%)	
Sex					
Men	295 (21.79)	306 (21.97)	287 (20.84)	212 (15.32)	
Women	1059 (78.21)	1087 (78.03)	1090 (79.16)	1172 (84.68)	< 0.001
Place of birth					
Mexico	1059 (78.21)	1079 (77.46)	1069 (77.63)	1092 (78.90)	
U.S	295 (21.79)	314 (22.54)	307 (22.29)	290 (20.95)	0.724
Insurance					
No	705 (52.07)	729 (52.33)	725 (52.69)	695 (50.22)	
Yes	649 947.93)	664 (47.67)	651 (47.31)	689 (49.78)	0.356
Physical activity					
Low	947 (70.94)	937 (68.10)	943 (69.39)	977 (71.47)	
Median	341 (25.54)	382 (27.76)	349 (25.68)	349 (25.53)	
High	47 (3.52)	57 (4.14)	67 (4.93)	41 (3.00)	0.276
Acculturation					
Low	838 (61.98)	885 (63.99)	859 (62.98)	897 (65.19)	
High	514 (38.02)	498 (36.01)	505 (37.02)	479 (34.81)	0.024

Pairwise correlation analysis was applied to assess the correlation among built environment variables (Table 2). We used Rho (ρ) ≥ 0.5 as the cutoff point to determine whether the correlation was significant. Significant correlations were observed between population density and household density (ρ = 0.9416), intersection density and road density (ρ = 0.9989), and walking time to the nearest park and networked distance to the nearest park (ρ = 0.9958).

**Table 2:** Pair-wise correlations between built environment variables.

	Population density	Household density	Intersection density	Road density	Networked distance to the park	Distance to highway	Walking time to the nearest park	Rundle's LUM	Frank's LUM	CDC mREFI
Population density	1.0000									
Household density	**0.9414**	1.0000								
Intersection density	**0.3025**	**0.3876**	1.0000							
Road density	**0.2994**	**0.3872**	**0.9989**	1.0000						
Networked distance to the park	**− 0.1091**	**− 0.0878**	**− 0.1476**	**− 0.1414**	1.0000					
Distance to highway	**0.1634**	**0.1334**	**− 0.2430**	**− 0.2300**	**0.0889**	1.0000				
Walking time to the nearest park	**− 0.1046**	**− 0.0823**	**− 0.1456**	**− 0.1394**	**0.9958**	**0.0882**	1.0000			
Rundle's LUM	**− 0.3641**	**− 0.2689**	**0.1559**	**0.1615**	0.0135	**− 0.1854**	0.0131	1.0000		
Frank's LUM	**− 0.4735**	**− 0.4089**	**− 0.0419**	**− 0.0355**	**− 0.1273**	**− 0.1627**	**− 0.1316**	**0.2754**	1.0000	
CDC mREFI	**0.082**	**0.0569**	**0.1518**	**0.1519**	**− 0.0353**	**0.0597**	**− 0.0302**	**0.0387**	**− 0.1104**	1.0000

All statistically significant correlations (P < 0.05) were in Bold.

To further evaluate the associations between individual built environment variable and telomere length, we used multinominal logistic regression analysis (Table 3). A significant association was observed between Rundle’s LUM and telomere length group. Using 1st quartile telomere length (shortest telomere length) as the reference group increased Rundle’s LUM was associated with increased odds of having high levels of telomere length (2nd quartile: OR 1.26, 95% CI 1.07, 1.48; 3rd quartile: OR 1.25, 95% CI 1.06, 1.46; 4th quartile: OR 1.19, 95% CI 1.01, 1.41, respectively). A similar association was also observed for Frank’s LUM. Increased Frank’s LUM was associated with elevated levels of telomere length (2nd quartile: OR 1.34, 95% CI 1.02, 2.63; 3rd quartile: OR 1.55, 95% CI 1.04, 2.91; 4th quartile: OR 1.36, 95% CI 1.05, 2.72, respectively). The covariates included age, sex, BMI, physical activity, health insurance, born place, acculturation, and census income. No significant association was observed for other built environment variables.

**Table 3:** Multinominal logistic regression to estimate the association between individual built environment variables and telomere length.

Variables	1st quartile	2nd quartile	3rd quartile	4th quartile
	ORs (95% CI)^a^	ORs (95% CI)^a^	ORs (95% CI)^a^	ORs (95% CI)^a^
Population density	1.00	0.94 (0.87, 1.02)	0.94 (0.86, 1.02)	0.99 (0.92, 1.08)
Household density	1.00	0.89 (0.71, 1.10)	0.83 (0.66, 1.04)	0.90 (0.72, 1.12)
Intersection density	1.00	0.97 (0.89, 1.04)	1.00 (0.92, 1.08)	1.01 (0.93, 1.09)
Road density	1.00	0.98 (0.94, 1.02)	1.00 (0.96, 1.04)	1.00 (0.96, 1.04)
Distance to highway	1.00	1.02 (0.94, 1.11)	0.96 (0.88, 1.05)	1.00 (0.91, 1.09)
Walking time to the nearest park	1.00	1.00 (0.99, 1.01)	1.00 (0.99, 1.01)	1.00 (0.99, 1.00)
Networked distance to the nearest park	1.00	1.03 (0.95, 1.15)	1.04 (0.94, 1.14)	0.94 (0.85, 1.04)
Rundle's LUM	1.00	1.26 (1.07, 1.48)	1.25 (1.06, 1.46)	1.19 (1.01, 1.41)
Frank's LUM	1.00	1.34 (1.02, 2.63)	1.55 (1.04, 2.91)	1.36 (1.05, 2.72)
CDC mREFI	1.00	1.00 (0.98, 1.01)	0.99 (0.97, 1.00)	1.00 (0.99, 1.02)

^a^Adjusted by age, sex, BMI, physical activity, health insurance, born place, acculturation, and census income.

Next, we selected seven non-redundant built environment variables and simultaneously included them in the multinominal logistic regression analysis (Table 4). Those variables were population density, intersection density, distance to highway, networked distance to the nearest park, Rundle’s LUM, Frank’s LUM, and CDC mRFEI. The only variable significantly associated with telomere length was still Rundle’s LUM. Increased Rundle’s LUM was associated with increased odds of having high levels of telomere length (2nd quartile: OR 1.27, 95% CI 1.06, 1.52; 3rd quartile: OR 1.23, 95% CI 1.03, 1.48; 4th quartile: OR 1.23, 95% CI 1.02, 1.48, respectively). The association between Frank’s LUM and telomere length was not statistically significant anymore.

**Table 4:** Multinominal logistic regression to estimate the association between non-redundant built environment variables and telomere length.

Variables	1st quartile	2nd quartile	3rd quartile	4th quartile
	ORs (95% CI)^a^	ORs (95% CI)^a^	ORs (95% CI)^a^	ORs (95% CI)^a^
Population density	1.00	1.00 (0.90, 1.11)	1.01 (0.91, 1.13)	1.04 (0.93, 1.16)
Intersection density	1.00	0.96 (0.88, 1.05)	1.00 (0.91, 1.09)	0.97 (0.89, 1.07)
Distance to highway	1.00	1.03 (0.94, 1.13)	0.99 (0.90, 1.08)	1.02 (0.92, 1.12)
Networked distance to the nearest park	1.00	1.01 (0.91, 1.13)	1.07 (0.96, 1.19)	0.96 (0.85, 1.07)
Rundle's LUM	1.00	1.27 (1.06, 1.52)	1.23 (1.03, 1.48)	1.23 (1.02, 1.48)
Frank's LUM	1.00	1.29 (0.95, 3.41)	1.24 (0.96, 3.12)	1.35 (0.92, 3.30)
CDC mREFI	1.00	1.00 (0.98, 1.02)	0.99 (0.97, 1.01)	1.01 (0.99, 1.02)

^a^Adjusted by age, sex, BMI, physical activity, health insurance, born place, acculturation, and census income.

Finally, we investigated whether the association between Rundle’s LUM and telomere length differed by demographic factors (Table 5). When stratified by age group, using median age of 38 years old, we found the association was evident among younger study participants (2nd quartile: OR 1.48, 95% CI 1.13, 1.93; 3rd quartile: OR 1.34, 95% CI 1.02, 1.76; 4th quartile: OR 1.23, 95% CI 0.94, 1.60, respectively), but not among older ones. The association was also only observed among women (2nd quartile: OR 1.37, 95% CI 1.13, 1.65; 3rd quartile: OR 1.32, 95% CI 1.09, 1.60; 4th quartile: OR 1.26, 95% CI 1.04, 1.54, respectively), but not among men. Also, we found the association was more evident among those with obesity, born in Mexico, having low levels of physical activity, and having low levels of acculturation than their relative counterparts.

**Table 5:** Association between rundle’s LUM and telomere length stratified by demographic variables.

Variables	1st quartile	2nd quartile	3rd quartile	4th quartile
	ORs (95% CI)^a^	ORs (95% CI)^a^	ORs (95% CI)^a^	ORs (95% CI)^a^
Age (by median)				
< 38 years old	1.00	1.48 (1.13, 1.93)	1.34 (1.02, 1.76)	1.23 (0.94, 1.60)
≥ 38 years old	1.00	1.15 (0.95, 1.39)	1.16 (0.96, 1.48)	1.13 (0.92, 1.39)
Sex				
Men	1.00	0.95 (0.68, 1.32)	1.04 (0.76, 1.43)	0.95 (0.64, 1.39)
Women	1.00	1.37 (1.13, 1.65)	1.32 (1.09, 1.60)	1.26 (1.04, 1.54)
BMI				
< 30	1.00	1.20 (0.96, 1.51)	1.23 (0.98, 1.55)	1.08 (0.84, 1.39)
≥ 30	1.00	1.32 (1.05, 1.65)	1.22 (0.97, 1.54)	1.27 (1.01, 1.61)
Born place				
Mexico	1.00	1.28 (1.07, 1.54)	1.23 (1.02, 1.48)	1.15 (0.94, 1.40)
U.S	1.00	1.11 (0.77, 1.59)	1.29 (0.91, 1.82)	1.29 (0.91, 1.85)
Physical activity				
Low	1.00	1.25 (1.04, 1.51)	1.22 (1.01, 1.48)	1.22 (1.01, 1.47)
Median and high	1.00	1.27 (0.92, 1.74)	1.26 (0.92, 1.73)	1.01 (0.70, 1.46)
Acculturation				
Low	1.00	1.34 (1.07, 1.69)	1.30 (1.03, 1.64)	1.29 (1.02, 1.62)
High	1.00	1.15 (0.85, 1.50)	1.16 (0.94, 1.43)	1.05 (0.82, 1.35)

^a^Adjusted by age, sex, BMI, physical activity, health insurance, born place, acculturation, and census income as appropriate.

## Discussion

Improved physical activity environment, diverse land use, and a healthy food environment, three significant domains of the built environment may promote healthy behaviors such as being physically active and eating healthy foods. On the other hand, living in a neighborhood with inadequate physical environment resources, less land use mixture, and an unhealthy food environment may increase chronic stress. Thus, built environment factors provide a noticeable and relevant point of an indication to the neighborhoods. In this cross-sectional study, we assessed the relationships between multiple components of the built environment with leukocyte telomere length, a well-regarded biomarker of biological aging, which can not only echo environmental exposure but also may also contribute to aging-related chronic diseases. A positive association was observed between Rundle’s LUM and telomere length. Moreover, the association was more evident among younger individuals (< 38 years old), women, and those with obesity, born in Mexico, having low levels of physical activity, and having low acculturation levels than their relative counterparts.

Our finding is in agreement with the idea that increased land use mixture positively impacts physical activity. Although the purpose of many urban planning patterns used in the last half-century has been to protect the public's health by separating industrial, commercial, and residential areas to improve quality of life, it may have had unintended costs^36,37^. Specifically, the unintentionally created residential environments are unfavorable to health because they are less supportive of physical activity, healthy eating, and sustainable living^38–40^. Moreover, it has been shown that diverse land use can create more enjoyable walking environments and promote physical activity^41^. In epidemiological studies, a positive association between physical activity and telomere lengths has been reported consistently in various populations^13,42–46^. Our previous study in the same study population observed that leukocyte telomere length decreased with sitting time^16^. Thus, our results here suggest that increased land use mixture may promote physical activity and thereby enhance the immune system and decrease telomere length shortening in leukocytes.

Unlike Rundle’s LUM, after adjusting with other built environment factors, the association between Frank’s LUM and telomere length was no longer significant. Different built environment features are included in two LUM indexes. The Rundle's LUM only considered two types of land use, including residential and commercial uses. In the area where our study participants reside, residential and commercial are the primary land uses. Besides residential and commercial uses, Frank's LUM also included farm ranch, industrial, parks, underdeveloped area, vacant area, etc. The inclusion of additional land use components may increase the mix but decrease the weight of significant land use, such as residential and commercial land use. This may explain why the weak association was observed between Frank’s LUM and leukocyte telomere length.

Lynch et al. reported that population density and more urban crowding were linked to shorter telomere length in certain telomere length groups^28^. We examined the association between population and household densities with telomere length in our study. Overall, no significant association was observed. The difference between the two studies may be due to the difference in population. Compared to Lynch’s study with 127 census tracts in three U.S. regions, our study population was limited to an area of Houston and its metropolitan area. Thus, our study may not have enough variation in the population and household densities and consequently limit our ability to detect the association. However, on the other side, Lynch’s study only included 1488 study subjects, which is much smaller than our study (N = 5508). In the future, more research is needed to further clarify the association between population density and telomere length.

Another interesting finding from this study is that the association between Rundle’s LUM and telomere length differs by demographic variables, including age group, gender, obesity status, levels of physical activity, and levels of acculturation. Compared to older individuals, younger individuals tend to be more active and involves in more outdoor activities. So, they are more likely to be affected by land use than their older counterparts. In our study population, women were more likely to have lower physical activity and being obese than men^10^. Better land use mixture may create an inviting environment for women to be more physically active. Because their starting level is already low, the health benefit from physical activity will be amplified. The same explanation can also describe why the association was more evident among individuals with obesity and physically inactive. The difference between acculturation levels is interesting. Our previous study has shown that acculturation was associated with a higher risk of obesity in U.S.-born participants and lower risk in Mexico-born participants^47^. Since the third fourth of our study participants was born in Mexico, the observed difference by acculturation may reflect the difference in obesity, which has been discussed above.

Our study has several limitations. First, our study design is cross-sectional, in which we cannot conclude the causal association between the exposure and outcome. Second, the demographic data used in our study were self-reported, which is subject to recall bias. Third, because of the study design in our original cohort, the study participants were recruited from areas known to be enriched have Mexican immigrants, which may lead to a relatively small variation in social economic status and built environment in neighborhoods. Thus, we must be very cautious to generalize our findings to other study populations.

In summary, our study is the first to evaluate the potential influence of the built environment on telomere length in Mexican Americans. Our results provide biological evidence to create a favorable built environment in urban and transportation planning that promotes active living. Such built environment may help slow down biological aging and ultimately improve the overall health. Future studies are needed to confirm the results of this study and further assess the relationship among built environment, telomere length, and chronic diseases.

## Data Availability

The datasets used and/or analyzed during the current study are available from the corresponding author on reasonable request.
